# Supplementary material for: Analysis of the Peritumoral Tissue Unveils Cellular Changes Associated with a High Risk of Recurrence
Source: Cancers (Basel). 2023 Jun 30;15(13):3450. doi: 10.3390/cancers15133450 (PMC10340275; doi:10.3390/cancers15133450)
Supplement: Supplementary file 1 [file cancers-15-03450-s001.zip › cancers-2425310-supplementary.pdf]

Supplementary Table S1 . Results of an immune score of principal inflammatory components corresponding to the global clustering presented in Figure 8 .

	M2	M0	Monocytes	Mast.cells.resting	Plasma.cells	B.cells.memory	T.cells.CD4.naive	Neutrophils	T.cells.follicular.he	M1	Tregs	NK.cells.resting	T.cells.CD4.memory	Dendritic.cells.resti	Dendritic.cells.acti	T.cells.gamma.delta	B.cells.naive	Eosinophils	Mast.cells.activate	T.cells.CD4.memory	T.cells.CD8	NK.cells.activated	GI : genomic index
8_R1	0.072430822	0.014124871	0.320189911	0	0.015749279	0.005358673	0.00846844	0.128394379	0.013316686	0.001382255	0.012968643	0	0	0	0.007966272	0	0	0	0.245395292	0.015098659	0.006860203	0.132295615	NA
20_HT	0.052666167	0	0.206615918	0	0.020227272	0.01144062	0	0.099333742	0.040444726	0.037689594	0	0	0	0	0.006049236	0	0	0	0.276265502	0.059654467	0.03945693	0.150155826	NA
1_R1	0.160023808	0.069997246	0.279319958	0	0.005627553	0.00174887	0	0.162833943	0	9.00E-04	0	0	0	0	0	0	0	0	0.176363963	0.101757184	0.009891991	0.031563506	NA
8_HT	0.035846109	0.072069769	0.301184452	0	0.056344173	0.125129229	0	0.068390555	0	0	0	0.067332479	0	0	0.006076408	0	0	0	0.097253772	0.104564421	0.049134429	0.016674203	NA
17_HT	0.093750494	0	0.273476343	0.011484737	0.234173356	0.018728734	0	0.041739118	0.027908236	0	0.092567209	0.047838045	0	0	0.014328396	0	0	0	0	0.055023254	0.053334893	0.035647185	NA
11_HT	0.109753203	0	0.275743083	0.066475112	0.201164959	0	0	0	0.076110595	0.003451739	0	0.024779119	0	0	0.004143695	0	0.005377513	0	0.034386662	0.087871496	0.034142181	0.076600644	NA
13_HT	0.186933736	0	0.29229634	0	0.137647147	0.040868258	0.047687762	0.034144024	0.0233398	0.003523593	0.014940788	0	0	0	8.00E-04	0	0	0	0.119830447	0	0.012971121	0.085062052	NA
7_HT	0.163774052	0	0.350484979	0.031782343	0.110254837	0.094842556	0	0.008897084	0	0	0	0	0	0	0.004376275	0	0	0	0.069206865	0.109253759	0.03066623	0.02646102	NA
1_HT	0	0.023839736	0.514650311	0	0.027040514	0.053450946	0.024715599	0	0.00556167	0	0.053626111	0	0	0	0.011204851	0	0	0	0.087070401	0.044649771	0.024093832	0.13009626	NA
19_HT	0.016140521	0.082749947	0.311066623	0.256509216	0.001313185	0.013619946	0	0.001010802	0.005383106	0	0.035659661	0	0	0	0.038730274	0	0	0	0.038897421	0.112402044	0.04697927	0.039537985	NA
6_T	0.04197721	0.492906856	0.032494976	0.016407287	0	0.049708486	0	0	0.044257907	0.011340067	0.022937976	0	0	0	0.007148579	0.046421762	0	0	0	0	0.16345951	0.070939385	17
13_T	0.356027658	0.533422275	0.002748008	0	0	0.015325175	0.003833875	0	0	0	0	0	0	0	0	0	0	0	0.039046411	0.034634626	0	0.014961972	114
11_T	0.185134283	0.513775624	0	0	0.039009549	0.103491892	0.029384979	0.03607056	0.010843074	0	0.01955796	0.017229538	0.011619218	0.003451546	0	0.007494464	0	0	0.015604569	0.007332744	0	0	158
17_T	0.346246533	0.119095358	0	0	0.029786803	0.069067886	0	0.025964992	0.030779958	0.010779361	0.009047122	0.02918709	0.014705615	0	0	0	0	0	0.171268644	0	0.144070637	0	385
2_T	0.4119461	0.269015599	0	0	0	0.005586164	0.020843986	0.002978761	0.071224612	0.017838963	0.001144298	0	0	0	0	0	0	0	0.12097856	0	0	0.078442958	277
10_T	0.271601389	0.265385682	0	0.03778118	0.101484201	0.093029275	0.017032957	0.005217464	0.020222482	0.018640336	0.048475718	0	0	0	0	0	0	0	0.036527705	0.008829895	0	0.075771716	134
20_T	0.301976551	0.287257013	0.027406507	0	4.00E-04	0.043939695	0	0	0	0.037943624	0	0	0.081714855	0	0	0	0	0	0.05419425	0.103906159	0.020265934	0.040989721	49
14_T	0.317876709	0.330073562	0.020604504	0	0.004513054	0.020214717	0	0.011475861	0.004474526	0.027351277	0.001	0	0	0	0	0	0	0	0.047006351	0	0.124633904	0.090796253	192
10_R1	0.419707079	0	0.045569678	0	0.022381014	0.029073008	0	0.00485489	0.027606557	0.023783681	0	0	0	0	0	0	0	0	0.215183019	0.073817618	0.027637283	0.110386174	NA
13_R1	0.363800881	0	0.11861146	0	0.001474317	0.00681853	0	0.002022908	0.015112806	0.047267683	0	0	0	0	0	0	0	0	0.246555086	0.098791384	0.016870943	0.082674001	NA
5_R1	0.365615659	0	0.156311379	0	0	0.019144849	0	0.04850545	0.002376601	0.006106641	0.001205916	0	0	0	0	0	0	0	0.152431495	0.10645099	0.027547828	0.114303192	NA
15_HT	0.318623245	0	0.074417474	0.045774447	0.053326313	0.007500983	0	0	0.016358017	0.025835495	0	0	0	0	0	0	0	0	0.130894879	0.090338358	0.07817763	0.158753158	NA
15_R1	0.330187568	0	0.089222324	0	0.029124704	0.029777954	0	0	4.00E-04	0.017059231	0	0	0	0	0.007731463	0	0	0	0.208986944	0.083377444	0.063947722	0.140195982	NA
18_T	0.370352739	0	0.079566155	0.038958616	0.006623238	0.012278898	0.145949875	0	7.00E-04	0.005755648	0.052170574	0.068946405	0	0.002968818	0	0	0	0	0	0.09459125	0	0.121137211	5
17_R1	0.457091918	0	0.064035241	0.023756617	0.010077771	0.025925087	0	9.00E-04	0.005060048	0.026246716	0	0	0	0	0	0	0	0	0.085087038	0.117490336	0.058249567	0.126112439	NA
12_HT	0.424340669	0	0.105277472	0.124680577	0.031727326	0.039210655	0	0	0	0	0	0	0	0	0	0	0	0	0	0.072040054	0.082964172	0.119759075	NA
11_R1	0.417102553	0	0.059727748	0.122369409	0.054596052	0	0	0	0.012609424	0.020133457	0	0	0	0	0	0	0.004261932	0	0.032855258	0.078123381	0.045831471	0.152389315	NA
6_R1	0.478738751	0	0.08484571	0.104081531	0.010588194	0.057736749	0	0	0.024920761	0.010208252	0.010110582	0	0	0	0.007896901	0	0	0	0	0.101002837	0.030207949	0.079661782	NA
9_R1	0.426587587	0.03352278	0.0464465	0.085370538	0.006493447	0.037127412	0	0	0.002994188	0.057926229	0	0	0	0	0	0.006074782	0	0	0	0.121720747	0.064505796	0.111229994	NA
7_R1	0.409618378	0	0.175027668	0.115530455	0.018841845	0.057783053	0.055721902	0	0	0.005518557	0.015673675	0	0	0	0	0	0	0	0	0.113905061	0	0.032379406	NA
7_T	0.42839347	0	0.249184695	0.069387284	0.005047239	0.029910724	0	0	0	0	0	0.02772518	0.07056217	0	0.030854844	0	0	0	0	0.030444293	0.058490102	0	138
4_R1	0.284576764	0	0.27186171	0.007065703	0.011951628	0.001297208	0.010470826	0	0.001116019	0.04420606	0.004130772	0	0	0	0	0	0	0.003369781	0.013415806	0.093835738	0.090315627	0.162386357	NA
4_HT	0.222176349	0	0.23400013	0.15993978	0.004142026	0.013483721	0	0.063732701	0.008296784	0.013630413	0	0	0	0	0	0	0	0	0	0.115840564	0.053101929	0.111655603	NA
5_T	0.271261313	0	0.161486426	0.058276019	0.008758079	0.118868415	0	0	0.030706828	0.046805611	0.020942935	0	0	0.009114062	0	0	0	0.003695873	0	0.110045711	0.073471701	0.086567027	109
4_T	0.25808695	0	0.133481349	0.017812423	0.008369444	0.028301677	0	0	0.002317289	0.067080251	0.039512929	0	0	0.005152682	0	0	0	0	0	0.130835656	0.199061561	0.10998779	65
15_T	0.411615564	0	0.115232668	0.110976744	0.005716844	0.008333485	0	0	0	0.016050391	0	0	0.054432313	0	0	0	0	0	0	0.037228783	0.160733243	0.079679964	16
9_T	0.261313465	0	0.098479705	0.138498404	0.013834177	0.045161708	0	0	0	0.019508675	0	0	0.112767662	0.004615951	0.005195308	0	0	0	0	0	0.206540008	0.094084938	97
5_HT	0.249125198	0	0.129790461	0.082881053	0.115768085	0.094306266	0.045814431	0.030431224	0.013154077	0.009223531	0.010062266	0	0	0	0	0	0	0	0	0.037178845	0.06593542	0.116329142	NA
16_T	0.265948051	0	0.084742686	0.148502227	0.049070381	0.121028186	0.072330146	0	0.00451446	0.071142347	0.048175131	0	0	0	0	0	0	0	0	0	0.021053563	0.113492822	127
3_T	0.270789264	0.143457371	0.062964701	0.142653226	0.017841278	0.057870918	0	0.010732125	0.005666325	0.027437164	0	0	0	0	0	0	0	0	0.002141138	0.093887519	0.063918722	0.100640247	49
10_R1	0.21719673	0.020448809	0.082008008	0.144185423	0.041878201	0.064301538	0	0.005562018	0	0.049204579	0	0	0	6.00E-04	0	0	0	0	0	0.144520461	0.128842575	0.101253296	NA
2_R1	0.342451565	0	0.014140321	0.166266773	0.005068251	0.04467797	0	0.003309286	0	0.00771178	0.026351599	0	0	0.106171741	0	0	0	0	0	0.156189718	0.071201615	0.056459381	NA
3_R1	0.249240906	0	0.113009311	0.176400955	0.005454507	0	0.06178094	0	0.031350657	0.0022393	0	0	0.02264036	0.05541264	0.060388963	0	0	0	0	0.083647739	0.105921903	0.032511818	NA
8_T	0.330215976	0	0.093047202	0.161703637	0.016820996	0.034757117	0	0	0	0.051812988	0.001273682	0	0	0.014486928	0	0.037431705	0	0	0	0.010401408	0.155102365	0.092945996	41
16_R1	0.282688656	0	0.112852728	0.195425271	0.080657136	0.039203192	0	0	0	0.020279187	0	0	0	0	0	0	0	0	0	0.062643902	0.118906422	0.087343506	NA
16_HT	0.354018162	0	0.142648979	0.209674092	0.03006448	0.020509123	0	0	0.004905662	0.019553845	0.009227957	0	0	0	0	0	0	0	0	0.035057863	0.113688463	0.060651374	NA
19_T	0.128977645	0	0.090770449	0	0	0.003053729	0	0	0.077819231	0	0	0	0	0	0.015712498	0	0	0	0.186251085	0	0.236174622	0.26124074	9
12_T	0.089853899	0.036770099	0.133879031	0	0.016740435	0.210585595	0	0	0.058679504	0.068423491	0.050297694	0	0	0	0	0	0	0	0.028888989	0.06257413	0.041664228	0.201642906	110
19_R1	0.098011189	0	0.188062252	0.026195337	0.051447179	0.043278505	0	0	0	0.009676616	0.004685323	0	0	0	0.002517874	0	0	0	0.152564727	0.192972403	0.067213552	0.163375042	NA
15_HT	0.098368115	0	0.247809411	0	0.01009131	0.021257203	0	0.04058961	0	0.038908298	0	0	0	0	0	0	0	0	0.089745935	0.161005528	0.114042294	0.178182296	NA
14_R1	0.201526271	0	0.196513609	0.027693644	0.106951582	0.03639615	0	0	0.014783821	0	0.022425008	0	0	0	0.010226202	0	0	0	0.09069366	0.061342642	0.075005314	0.156442098	NA
18_HT	0.151613507	0	0.161215305	0	0.086647158	0.059189117	0	0	0.013092781	0.007790857	0.017122425	0	0	0	1.00E-04	0	0	0	0.178094407	0.101363091	0.087361353	0.136455759	NA
6_HT	0.088409164	0	0.168974358	0	0.153105164	0.055065035	0	0.026614103	0.033169468	0.033137497	0	0	0	0	0.003861309	0	0	0	0.159726295	0.066194185	0.048534633	0.163208788	NA
1_T	0.167077109	0.019792426	0.140010325	0.080643999	0.01015849	0.042843662	0.180146332	0	0	0	0.034783112	0.060663002	0	0.005711793	0	0	0	0.003854558	0	0.245619839	0	0.008695352	61
14_HT	0.153298507	0	0.086535138	0.14579862	0.115782154	0	0	0	0.067826198	0.044736325	0.049697202	0.147880632	0	0	0	0	0.004170964	0	0	0.163370407	0.020903854	0	NA
12_R1	0.234234572	0.033569363	0.055964938	0.369979174	0	0.085252797	0.052017408	0	0.014584618	0.006171827	0.0105753	0	0	0.009696566	0	0	0	0	0	0.015524018	0.056757341	0.055672079	NA
3_HT	0.226615141	0	0.050348746	0.334805218	0.020484305	0.047235134	0	4.00E-04	0.024518014	0.017451763	0	0	0	0.035424986	0.002083568	0	0	0	0	0.113003582	0.07148619	0.056122625	NA
18_R1	0.197819472	0.005610092	0.214195894	0.164474761	0.110754296	0.088545923	0.020547865	0	0	0	0.025416471	0	0	0	0	0	0	0	0	0	0.092385536	0.080249689	NA
2_HT	0.167278117	0	0.148670287	0.270616517	0.148380306	0.046320422	0	0	0.012334798	0.005829137	0.013759224	0	0	0	0	0	0	0	0	0	0.103329076	0.083482117	NA
10_HT	0.196979576	0	0.111573544	0.195825086	0.146566927	0.075478684	0	0	0.032895319	0.007379653	6.00E-04	0	0	0	0	0	0	0	0	0.068265243	0.066877494	0.097514517	NA

Supplementary Table S2: Comparison of genomic index tumors from R1.h group vs R1.t group.

Characteristic	0, N=8[1]	1, N=12[1]	p-value[2]
Genomic index	87.75	120.9	0.42
[1] Mean (SD)			
[2] Welch Two Sample t-test			

Supplementary Figure S1: Penetrance plot of data from CGH.

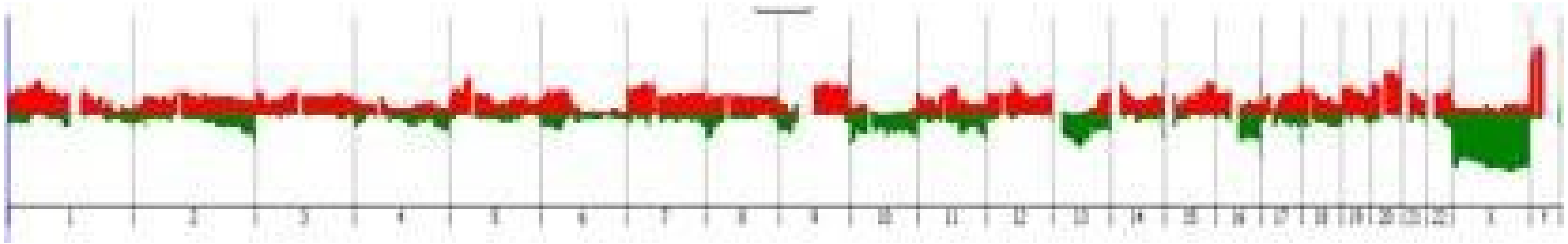

Supplementary Figure S2: Genomic profiles of other cases obtained by CGH.data 2

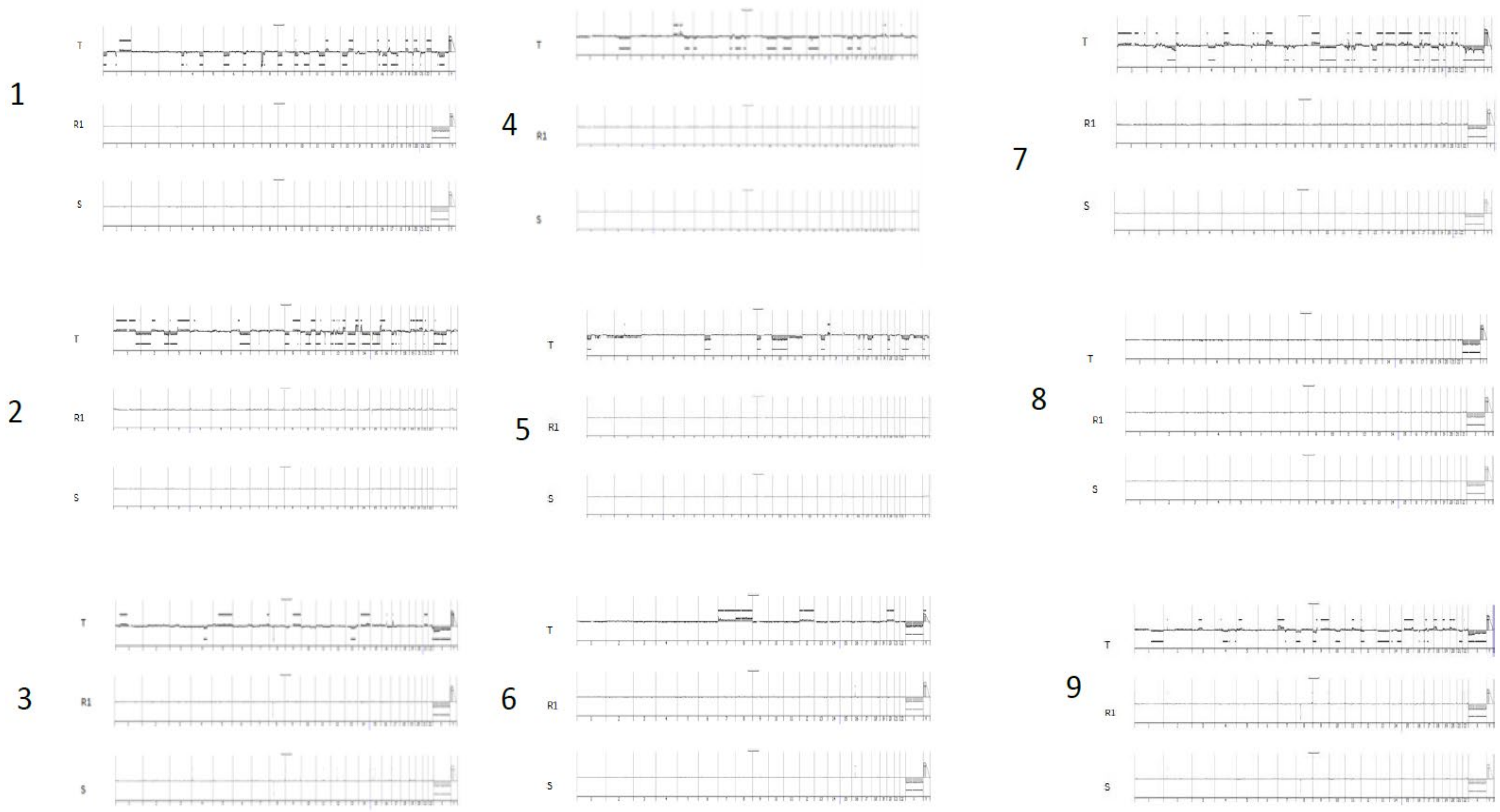

10

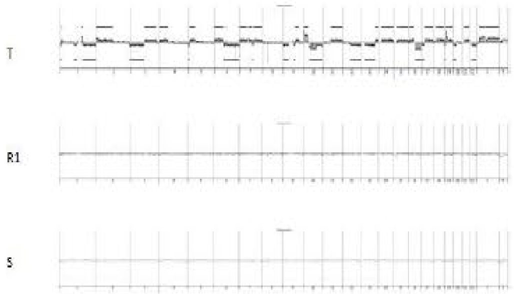

11

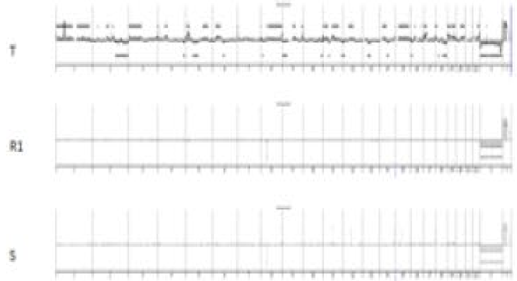

12

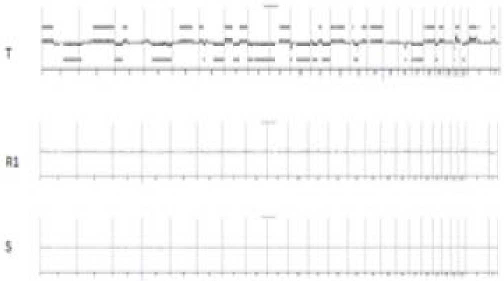

13

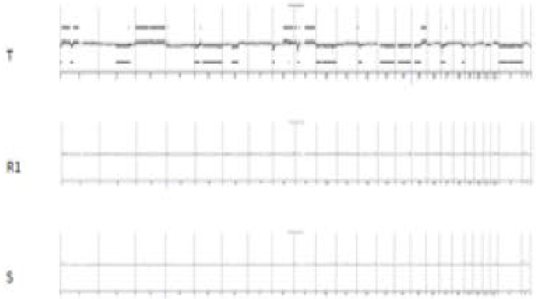

14

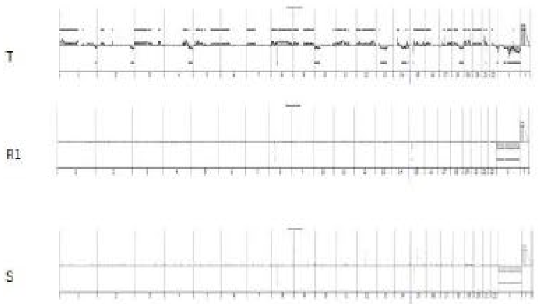

15

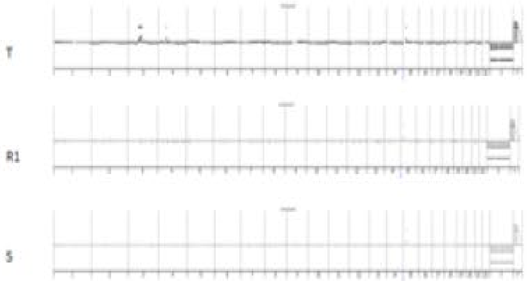

16

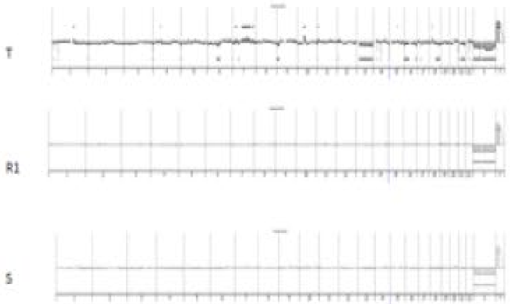

17

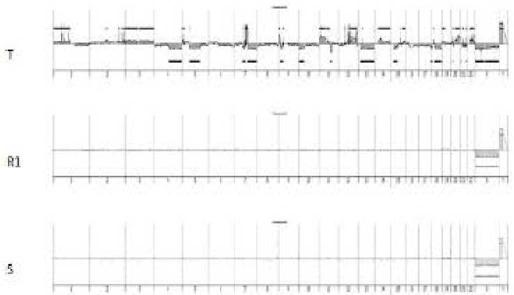

18

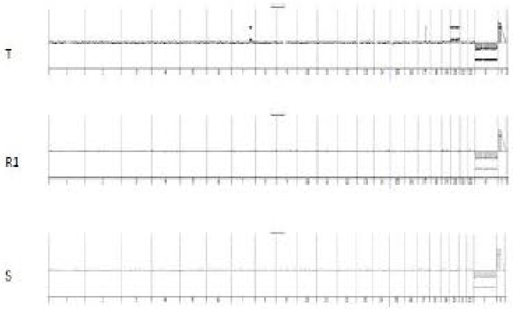

19

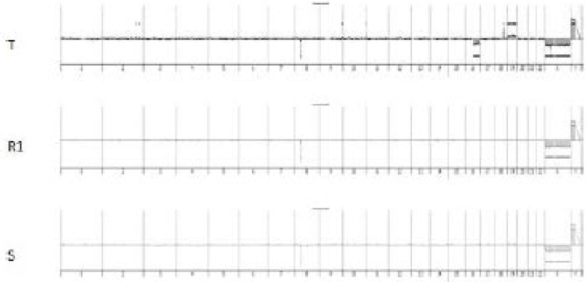

20

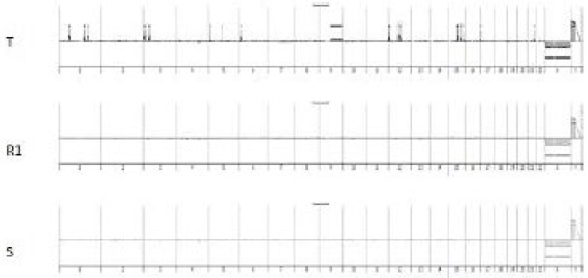

Supplementary Figure S3: Box plot of data of CIBERSORT between R1.h group vs R1.t group.

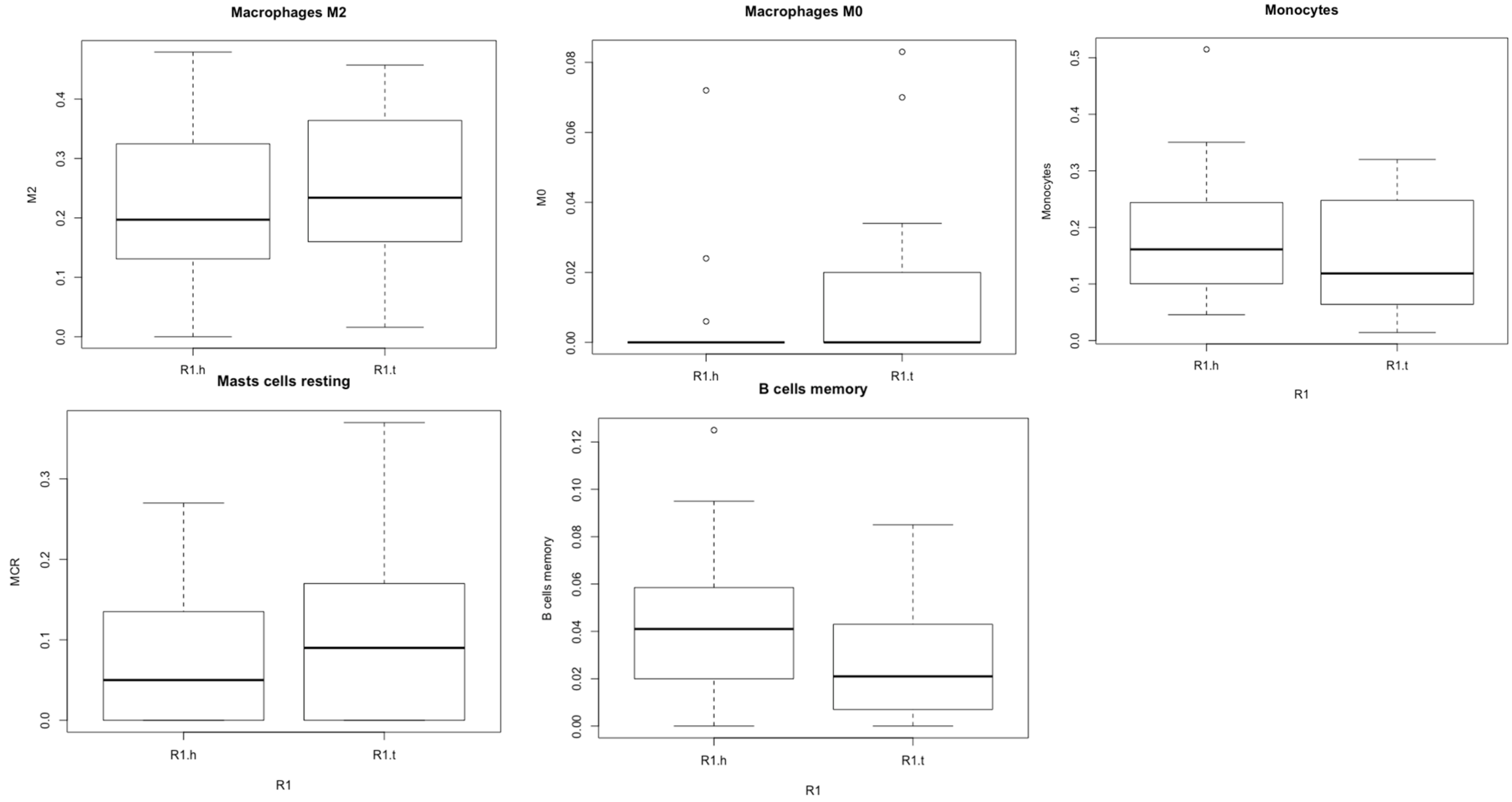

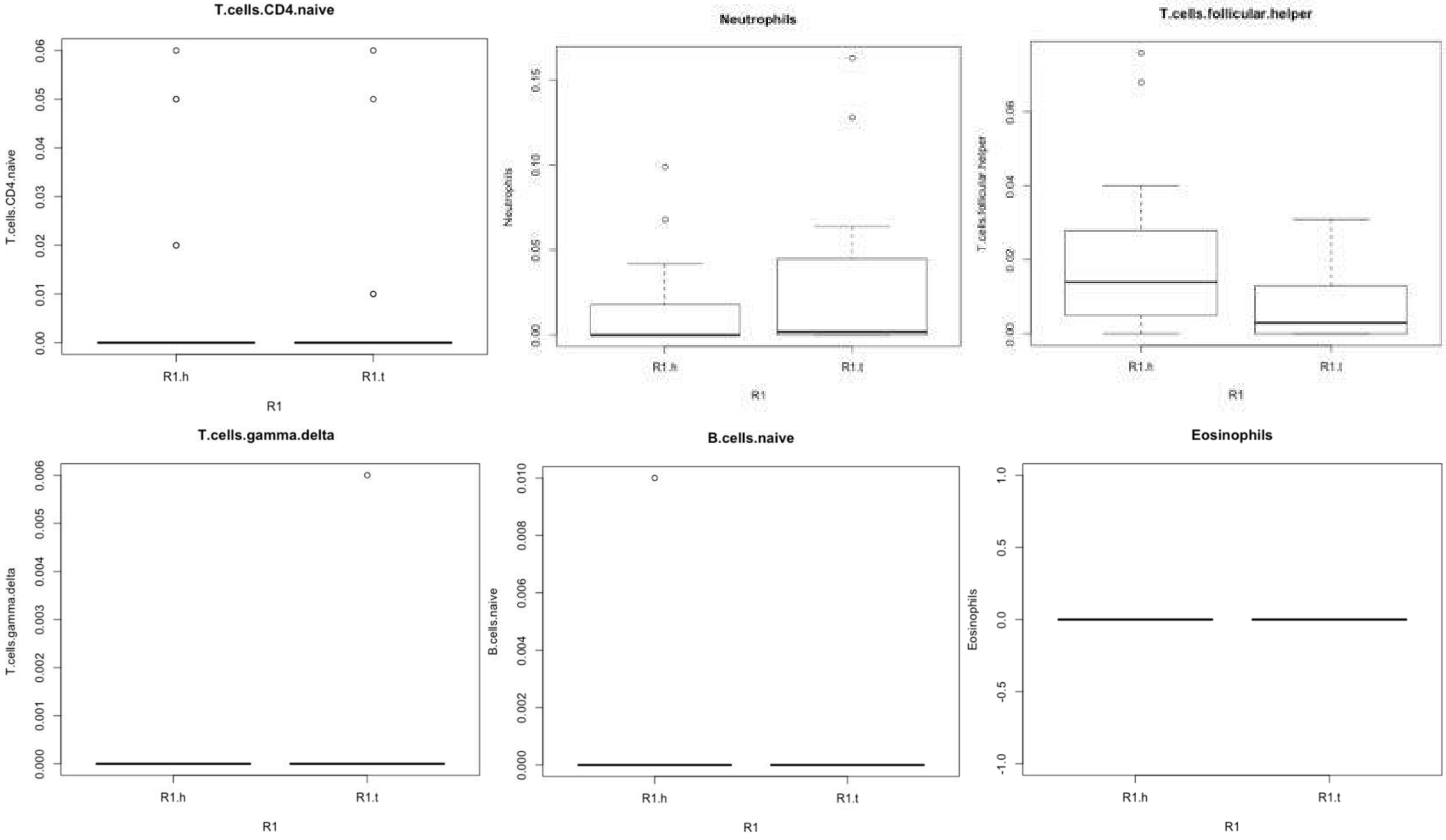
T.cells.CD4.naive
Neutrophils
T.cells.follicular.helper
T.cells.gamma.delta
B.cells.naive
Eosinophils
R1.h
R1.t
R1

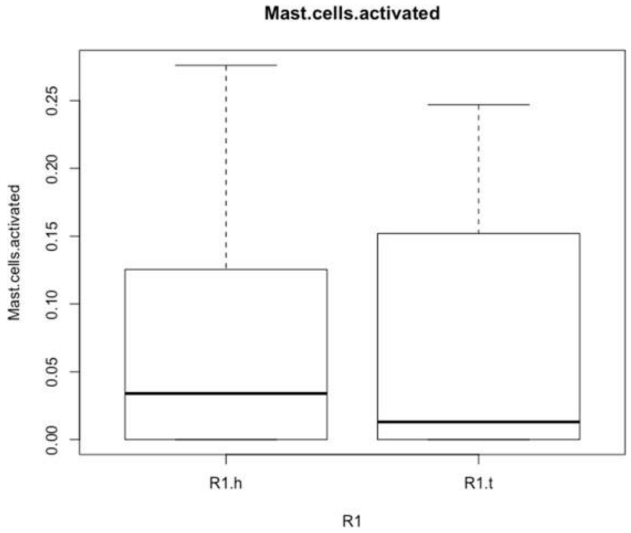
Mast.cells.activated
Mast.cells.activated
0.00
0.05
0.10
0.15
0.20
0.25
R1.h
R1.t
R1

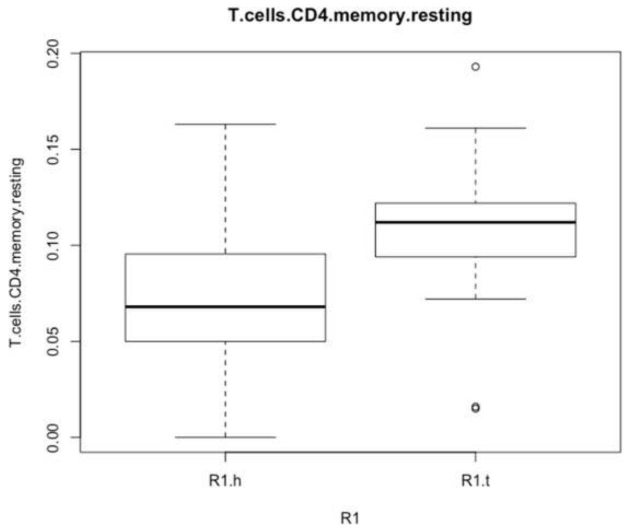
T.cells.CD4.memory.resting
T.cells.CD4.memory.resting
0.00
0.05
0.10
0.15
0.20
R1.h
R1.t
R1

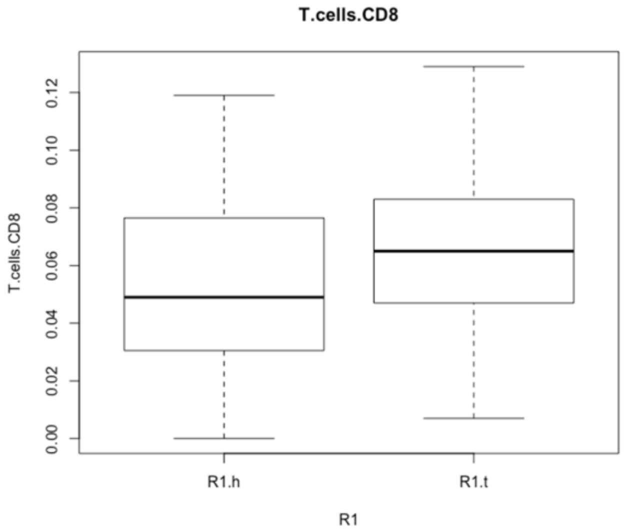
T.cells.CD8
T.cells.CD8
0.00
0.02
0.04
0.06
0.08
0.10
0.12
R1.h
R1.t
R1

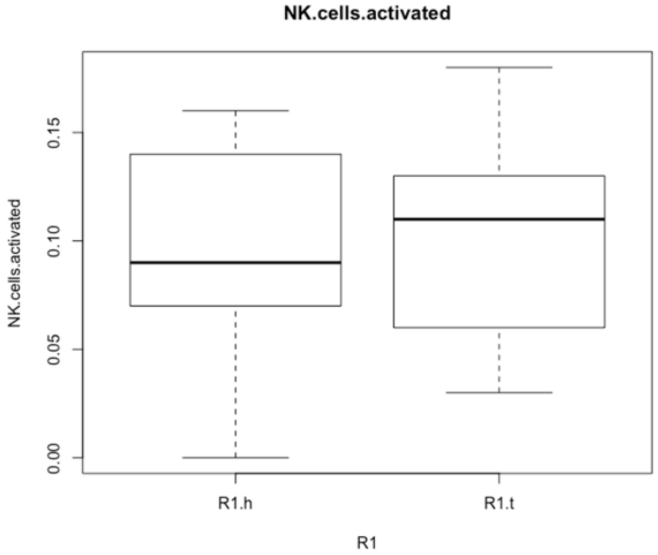
NK.cells.activated
NK.cells.activated
0.00
0.05
0.10
0.15
R1.h
R1.t
R1
